# Clinical Evaluation of ChatGPT-5.3 Responses to Patient-Oriented Questions on Scoliosis: A Multidimensional Expert Analysis

**DOI:** 10.3390/healthcare14111563

**Published:** 2026-06-03

**Authors:** Muhsin Doran

**Affiliations:** Department of Physical Medicine and Rehabilitation, Liv Hospital Vadistanbul, Ayazağa Mahallesi, Kemerburgaz Caddesi, Vadistanbul Park Etabı, 7F Blok, 34475 Istanbul, Turkey; muhsin-doran@hotmail.com; Tel.: +90-534-080-7940

**Keywords:** ChatGPT-5.3, large language models, patient education, scoliosis, clinical accuracy

## Abstract

**Highlights:**

**What are the main findings?**

- ChatGPT-5.3 produced clinically acceptable and highly understandable responses to scoliosis-related patient questions, with consistently high expert-rated content appropriateness.
- No high-risk outputs were identified; however, a subset of responses lacked sufficient clinical depth.

**What are the implications of the main findings?**

- ChatGPT-5.3 demonstrates strong potential as a scalable tool for patient education in musculoskeletal conditions.
- Despite its strengths, AI-generated responses require clinical oversight and should not replace professional medical judgment.

**Abstract:**

**Background:** The integration of large language models (LLMs) into healthcare has rapidly expanded, particularly in the domain of patient education. However, concerns remain regarding the accuracy, adequacy, and clinical safety of AI-generated medical information. This study aimed to systematically evaluate expert-perceived quality, content appropriateness, and potential clinical risk of responses generated by a GPT-5.3–based large language model to patient-oriented questions on scoliosis. **Methods:** A set of patient-oriented questions was developed based on common informational needs. Responses generated by the AI model were evaluated by a panel of experts using a multidimensional assessment framework, including general appropriateness, scientific accuracy, adequacy, and clarity. Content validity was assessed using the Content Validity Ratio (CVR) and Content Validity Index (CVI). In addition, clinical risk levels were categorized, and common issues in inappropriate responses were analyzed. CVR and CVI were used to quantify expert agreement regarding perceived content appropriateness, rather than to establish definitive factual correctness or guideline concordance. **Results:** All responses exceeded the predefined acceptable CVR threshold, and overall CVI values were high, indicating a high level of expert agreement regarding perceived response appropriateness. Clarity received the highest scores across all dimensions, whereas adequacy and scientific accuracy were relatively lower. Most responses were classified as harmless or low risk, and no high-risk responses were identified. The most frequently reported issue in inappropriate responses was insufficient information. **Conclusions:** Large language models may provide understandable and generally acceptable responses in a controlled expert-evaluation setting. However, these findings reflect expert-perceived appropriateness rather than definitive clinical validity or guideline concordance. AI-generated responses should therefore be used only as supportive educational tools under clinical oversight.

## 1. Introduction

The integration of artificial intelligence (AI) in medicine has long been a subject of discussion; however, a notable increase in clinical studies and investments in this field has been observed in recent years [1,2]. These technologies, which are increasingly being utilized in diagnosis, treatment planning, and clinical decision support systems, have significantly transformed the ways in which information is accessed and used in healthcare [2,3].

Large language models (LLMs), as a key component of these advancements, have attracted considerable attention due to their ability to generate instant and structured responses to natural language queries [4,5]. Systems such as ChatGPT and Gemini have emerged as alternative sources of medical information not only for healthcare professionals but also for patients, owing to their ability to provide rapid access to medical knowledge [1,6]. This has brought into focus the potential role of these systems, particularly in areas where health literacy and patient education are of increasing importance [6,7].

Nevertheless, the role of AI applications in clinical practice has not developed uniformly across all disciplines, and the number of studies remains relatively limited, particularly in the fields of rehabilitation and musculoskeletal disorders [8,9]

Adolescent idiopathic scoliosis (AIS) is a commonly encountered spinal deformity in clinical practice that requires long-term follow-up. The success of conservative treatment approaches largely depends on the patient’s understanding of the condition and active participation in the treatment process [10,11]. Today, patients and their families frequently seek health-related information via the internet and increasingly turn to AI-based systems during this process [1,6]. Moreover, the fact that scoliosis predominantly affects adolescents is noteworthy, as this population demonstrates high engagement with digital technologies and a strong capacity to adapt to innovative technological tools.

In this context, LLMs may serve as a potential source of information in patient education processes [6,12]. The growing tendency of young individuals to rely on digital resources and AI-based tools for health-related inquiries necessitates the evaluation of the accuracy, reliability, and clinical appropriateness of the information provided by these systems [1,13].

Although existing studies indicate a growing body of evidence regarding the performance of LLMs across various medical domains [1,2], there remains a limited number of studies that comprehensively evaluate responses to patient-oriented queries specifically in the context of scoliosis [8,14,15]. Furthermore, these responses should be assessed not only in terms of accuracy but also in terms of readability and patient-centered appropriateness [6,13]. While AI-based LLMs offer potential benefits in patient education, they must be carefully evaluated due to the risk of generating inaccurate or incomplete information [1,13].

This study aimed to evaluate ChatGPT-5.3 (OpenAI, San Francisco, CA, USA; 2026) responses to patient-oriented questions on adolescent idiopathic scoliosis in terms of expert-perceived appropriateness, scientific accuracy, adequacy, clarity, and potential clinical risk.

## 2. Materials and Methods

### 2.1. Study Design and Ethical Considerations

This study was designed as a cross-sectional expert evaluation study aimed at assessing the content validity and quality characteristics of responses provided to patient-oriented questions regarding the conservative management of idiopathic scoliosis. The study process consisted of two main phases: (1) the development of a question set reflecting the patient perspective, and (2) the multidimensional evaluation of AI-generated responses to these questions by expert clinicians.

This study did not involve patients, patient records, biological samples, clinical interventions, or identifiable patient data. The study was based solely on an anonymous voluntary survey completed by expert clinicians who evaluated AI-generated educational content. No identifiable or sensitive personal data were collected. Electronic informed consent was obtained from all expert participants before participation.

Because the study did not involve patients, clinical interventions, biological materials, or identifiable health data, formal ethical approval was not required according to the applicable national regulatory framework [16]. Nevertheless, the study was conducted in accordance with the ethical principles of voluntary participation, anonymity, confidentiality, and responsible research conduct. The reporting of the study was also informed by the DECIDE-AI framework for the early-stage evaluation of artificial intelligence–based clinical decision support systems [17].

### 2.2. Development of the Question Set

The questions used in this study were determined through a systematic content review to represent the most frequently asked topics by patients in routine clinical practice regarding scoliosis. For this purpose, authoritative national and international websites were examined, including the Turkish Scoliosis Research and Treatment Society (https://skolyozdernegi.com/, accessed on 30 January 2026), the Scoliosis Research Society (https://www.srs.org/, accessed on 30 January 2026), the International Society on Scoliosis Orthopaedic and Rehabilitation Treatment (SOSORT) (https://sosort.org/, accessed on 30 January 2026), and the National Scoliosis Foundation (https://www.scoliosis.org/, accessed on 30 January 2026).

In addition, a Google search was conducted to identify commonly accessed patient-oriented information. To ensure standardization and minimize potential bias, the search was performed on 30 January 2026, using incognito mode with a cleared browser cache and no active user account to avoid personalization effects. All searches were conducted in Turkish using the keyword “skolyoz,” with location settings fixed to Türkiye. The first 10 non-sponsored results were included, while advertisements and promoted content were excluded from the analysis.

The data obtained from these sources were combined to create a comprehensive pool of questions covering the most frequently expressed concerns of patients. The question pool was reviewed in terms of clinical relevance, content coverage, and clarity. Duplicate or conceptually overlapping questions were removed, and the final set of questions was established. As a result, a total of 20 questions were included in the study, covering key aspects of the condition, including definition, etiology, progression, diagnostic methods, conservative treatment approaches, and impact on daily life (Table 1).

All survey materials and evaluations were conducted in Turkish to ensure consistency with the language of the search and the target patient population.

Questions were not intended to represent an exhaustive or validated inventory of all patient informational needs. Instead, they were designed to capture common patient-oriented themes encountered in publicly accessible scoliosis information sources. The final set was reviewed for thematic coverage, clinical relevance, clarity, and avoidance of conceptual overlap.

### 2.3. Generation of AI Responses

The selected questions were submitted to a large language model (ChatGPT-5.3) on the same day using a standardized approach. To ensure consistency and reflect a patient-centered perspective, all questions were initiated with the phrase “I am a patient with scoliosis.”

Additionally, to ensure comparability in terms of length and content, the model was instructed to generate responses between 150 and 200 words for each question. The generated responses were recorded verbatim without any modifications and compiled in a standardized format for evaluation.

To prevent interaction effects between responses, each question was submitted in a separate session by opening a new ChatGPT interface.

### 2.4. Expert Panel

An expert panel consisting of healthcare professionals from different disciplines was formed to evaluate the responses. The panel included clinicians with experience in physical medicine and rehabilitation, orthopedics and traumatology, and physiotherapy. All participants were required to have clinical experience in the evaluation and management of patients with scoliosis.

### 2.5. Evaluation Procedure

An online evaluation form (Google Forms) containing all AI-generated responses was distributed to the experts. Each response was evaluated independently and anonymously.

Experts were asked to assess each response across four dimensions:

Overall appropriateness
Scientific accuracy
Adequacy (content coverage)
Clarity (understandability)

For reporting clarity, each evaluation dimension was operationally defined. Overall appropriateness referred to the suitability of the response for patient education in a clinical context. Scientific accuracy referred to consistency with current accepted medical knowledge. Adequacy referred to whether the response sufficiently covered the key aspects of the question. Clarity referred to the understandability of the response for a patient audience.

All evaluations were performed using a 6-point Likert scale:

1: Completely inappropriate
2: Inappropriate
3: Partially inappropriate
4: Partially appropriate
5: Appropriate
6: Completely appropriate

For content validity analysis, scores were dichotomized:

1–3: Inappropriate
4–6: Appropriate

For CVR/CVI calculation, Likert scores were dichotomized as 1–3 = inappropriate and 4–6 = appropriate, in accordance with the predefined content validity framework. This dichotomization was used only for CVR/CVI analyses. The original 6-point ordinal ratings were retained for descriptive analyses and sensitivity analyses.

For responses rated as “inappropriate,” experts were asked to specify the reason. The following predefined categories were used (multiple selections were allowed, with optional additional comments):

Contains scientific errors
Contains incomplete information
Does not adequately answer the question/off-topic
Language or expression issues
Contains excessive or unnecessary information
Other (with open-ended explanation)

### 2.6. Clinical Risk Assessment

Clinical risk was evaluated based on the potential of each response to cause patient misguidance, delayed care, or inappropriate self-management. Risk levels were classified as harmless, low risk, moderate risk, and high risk according to the operational definitions presented in Table 2:

At the end of the survey, experts were also asked to provide overall evaluations regarding:

The general quality of ChatGPT responses
Their usability in patient education
Their appropriateness for use without physician supervision
Commonly encountered issues

### 2.7. Statistical Analysis

All statistical analyses were performed using IBM SPSS Statistics for Windows, Version 31.0 (IBM Corp., Armonk, NY, USA). Ordinal data were presented as median and interquartile range (IQR), while categorical data were expressed as frequency and percentage (%).

Content validity was assessed using the Content Validity Ratio (CVR). CVR was calculated using the following formula:

CVR = (n_e_ − N/2)/(N/2)

where “n_e_” represents the number of experts rating a response as “appropriate,” and N represents the total number of experts.

The Content Validity Index (CVI) was calculated as the average of CVR values across all questions.

Considering the number of experts (N = 51), the minimum acceptable CVR threshold was determined to be approximately 0.26 according to Lawshe’s criteria, and values above this threshold were considered acceptable in terms of content validity [18].

The primary analysis was conducted based on the “overall appropriateness” dimension. Additionally, CVR values were calculated separately for scientific accuracy, adequacy, and clarity to provide a detailed evaluation of responses across different quality dimensions.

Reasons for inadequate or inappropriate responses were analyzed as categorical variables, and their frequency and percentage distributions were calculated. The chi-square test was used to compare the distribution of inadequacy reasons across different questions.

Inter-rater agreement was assessed using Fleiss’ kappa in IBM SPSS Statistics for Windows, version 26.0 (IBM Corp., Armonk, NY, USA; 2019).

## 3. Results

A total of 51 experts participated in the study. Of these, 52.9% were female and 47.1% were male. The mean age was 37.8 ± 7.4 years, and the mean professional experience was 12.5 ± 7.7 years. Participants were from the fields of Physical Medicine and Rehabilitation (47.1%), Orthopedics and Traumatology (29.4%), and Physiotherapy (23.5%). All participants had experience in the assessment and treatment of scoliosis (Table 3).

Fleiss’ kappa for the primary evaluation dimension, general appropriateness, was 0.138.

Table 4 presents the item-level appropriateness rates and CVR values for all evaluated questions across the four assessment dimensions. The CVR/CVI analysis demonstrated a high level of expert agreement across all evaluated dimensions. CVR values met or exceeded the predefined acceptable threshold for all questions, with most items achieving values close to 1.00. The proportion of responses rated as appropriate ranged from 94.1% to 100.0%.

Relatively lower CVR values were observed in a limited number of questions, particularly for Q18, which addressed the most suitable sport for scoliosis. For this item, scientific accuracy and adequacy showed the lowest agreement levels, with appropriateness rates of 94.1% and CVR values of 0.88. Minor dimension-specific variations were also observed in selected items, including Q2, Q6, Q7, Q8, Q12, Q16, and Q20; however, overall expert agreement remained high.

The overall Content Validity Index (CVI) was 0.99 for general appropriateness and clarity, and 0.98 for scientific accuracy and adequacy. These findings indicate a high level of expert agreement regarding the perceived appropriateness of the AI-generated responses across the evaluated dimensions.

As a sensitivity analysis, the original 6-point ordinal ratings were analyzed without dichotomization. Mean scores remained high across all evaluation dimensions, ranging from 5.03 ± 1.01 for adequacy to 5.17 ± 0.99 for clarity. The median score was 6.0 across all dimensions, with an IQR of 2.0, indicating that the overall rating pattern remained favorable when the full ordinal scale was considered (Table 5).

Analysis of the reasons for inappropriate ratings in general appropriateness revealed that the most common issue was insufficient information (52.2%), followed by scientific inaccuracies (21.7%), language or clarity issues (8.7%), excessive or unnecessary information (8.7%), and failure to adequately answer the question (4.3%) (Table 6).

**Figure 1:**
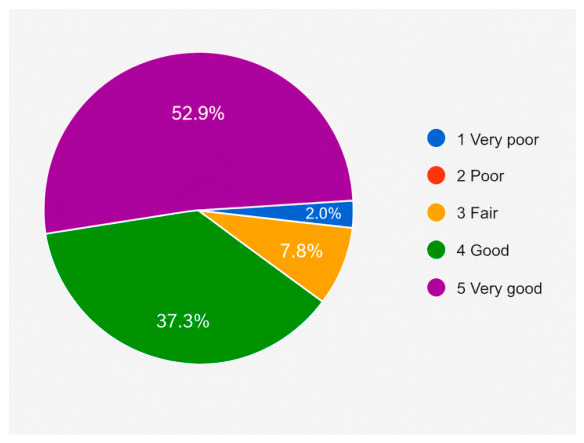
Expert general evaluation of ChatGPT responses on scoliosis (n = 51).

**Figure 2:**
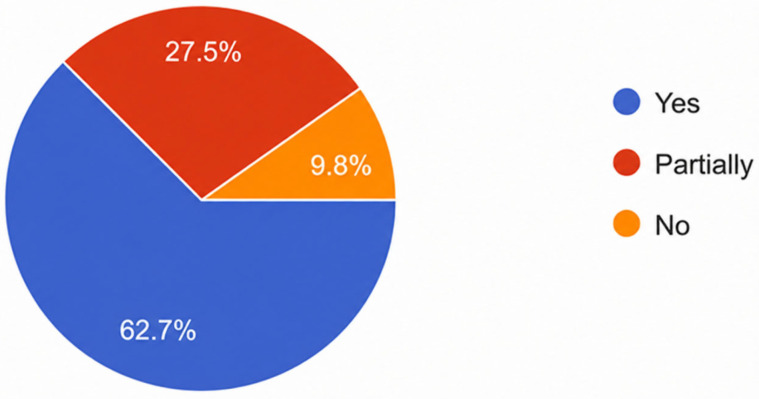
Expert opinions on the usefulness of AI-generated responses for patient education(n = 51).

Expert opinions regarding the overall evaluation and educational usefulness of ChatGPT-generated responses are presented in Figure 1 and Figure 2. Overall, 27 experts (52.9%) rated the responses as very good, 19 (37.3%) as good, 4 (7.8%) as fair, and 1 (2.0%) as very poor. Regarding usefulness for patient education, 32 experts (62.7%) answered “yes,” 14 (27.5%) answered “partially,” and 5 (9.8%) answered “no.”

**Figure 3:**
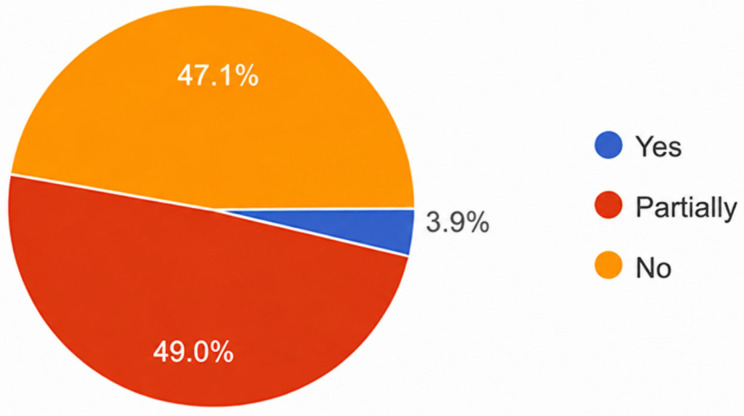
Expert opinions on the appropriateness of presenting AI-generated responses to patients without physician supervision (n = 51).

However, experts were more cautious regarding use without physician control. As shown in Figure 3, only 2 experts (3.9%) considered it appropriate to present AI-generated responses to patients without physician control, whereas 25 (49.0%) answered “partially” and 24 (47.1%) answered “no.”

**Figure 4:**
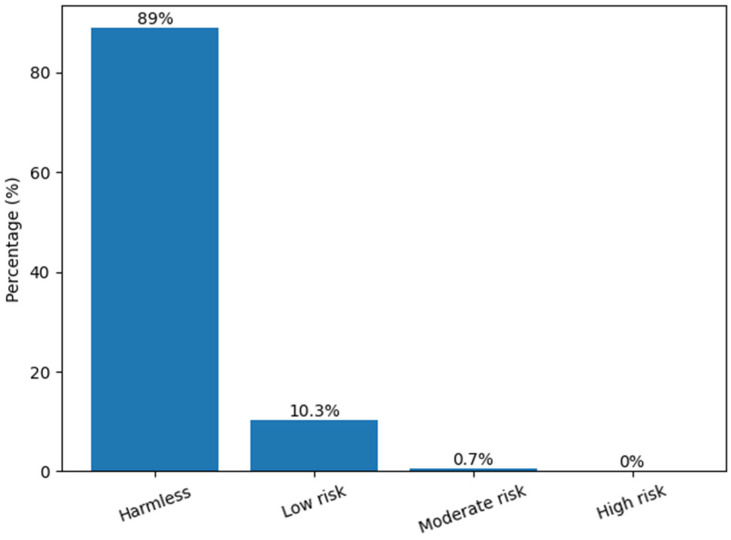
Clinical Risk Distribution of AI-Generated Responses According to Experts (Q1–20).

In terms of clinical risk, the majority of responses were classified as harmless or low risk, while a smaller proportion were categorized as moderate risk (Figure 4). No responses were classified as high risk. Risk percentages were calculated across all expert-response ratings (20 questions × 51 experts = 1020 ratings)

The most frequently reported issue was insufficient information (47.1%), while no issues were identified in 35.3% of responses. Other reported problems included incomplete or off-topic responses (7.8%), excessive information (5.9%), and scientific errors (3.9%) (Table 7).

## 4. Discussion

In this study, responses generated by a GPT-5.3–based large language model to patient-oriented questions on scoliosis were analyzed using a multidimensional expert evaluation approach. The findings indicate that, in this controlled expert-evaluation setting, ChatGPT-5.3 responses were generally perceived by clinicians as appropriate and understandable for patient education; however, such responses should still be carefully interpreted in terms of factual accuracy, content adequacy, and clinical safety.

Compared with previous studies evaluating earlier versions of large language models, our findings showed higher CVR/CVI values and higher levels of expert-rated appropriateness [15]. For instance, a recent study evaluating ChatGPT-4.0 in the context of scoliosis reported that 78.5% of responses met the minimum CVR threshold, with an overall CVI of 0.68, and several responses showed limitations in scientific accuracy and completeness [15]. In contrast, in the present study, all responses exceeded the predefined CVR threshold, and overall CVI values approached 0.99 across multiple evaluation domains. This difference may partly reflect improvements in large language model performance over time; however, it may also be related to differences in question selection, prompt structure, language, expert panel composition, scoring framework, and statistical handling of ratings. Therefore, direct version-based superiority cannot be inferred.

These findings are specific to ChatGPT-5.3 under the standardized prompting conditions used in this study. Patients may use different or freely accessible LLM versions in real-world settings, and these versions may vary in accuracy, completeness, safety safeguards, and response style. Therefore, the present findings should not be generalized to all AI-generated scoliosis information or interpreted as supporting independent patient use. Regardless of the source of differences between models, the persistence of insufficient detail in some responses indicates that professional contextualization remains important. Importantly, unlike previous studies, our analysis also incorporated clinical risk assessment, thereby providing additional descriptive insight into the perceived safety profile of AI-generated responses.

From an expert-agreement perspective, all questions exceeded the predefined CVR threshold, and the overall CVI values were high, indicating that the responses were generally perceived as appropriate by the expert panel. These findings are consistent with previous studies suggesting that large language models may generate medical information that is often perceived as understandable and useful, although concerns regarding completeness, contextual accuracy, and safety remain. Prior research has reported promising performance of large language models in medical knowledge tasks and clinical question-answering contexts [19,20,21]. Importantly, high CVR and CVI values in this study should be interpreted as indicators of expert agreement regarding perceived appropriateness, not as definitive evidence of factual correctness, clinical reliability, or guideline concordance.

Although the overall appropriateness rates and CVR/CVI values were high, Fleiss’ kappa for the primary evaluation dimension indicated only slight agreement. This apparent discrepancy should be interpreted in light of the marked ceiling effect and substantial category imbalance in the rating distribution. Because most responses were rated as appropriate, kappa statistics may underestimate agreement despite high observed concordance. This pattern is consistent with the known kappa paradox and suggests that Fleiss’ kappa should not be interpreted in isolation in datasets with highly imbalanced ratings [22].

However, evaluations based solely on overall appropriateness may be insufficient to capture all aspects of response quality. For this reason, responses in the present study were additionally assessed in terms of scientific accuracy, adequacy, and clarity. The results demonstrated that clarity received the highest scores, whereas adequacy and scientific accuracy were relatively lower. This suggests that while large language models are capable of generating generally appropriate and fluent content, some responses may remain limited in terms of depth and clinical detail.

Analysis of responses rated as inappropriate revealed that the most common issue was insufficient information. This suggests that the main limitation of the AI-generated responses was not necessarily overtly incorrect information, but rather limited depth or insufficient clinical contextualization. This may be particularly relevant for questions requiring individualized guidance, such as sports participation, bracing, treatment selection, follow-up, or specialist referral. Nevertheless, the overall findings suggest that the evaluated model was generally rated by experts as producing understandable and patient-oriented responses. The high proportion of responses rated as appropriate supports the potential use of such systems as supportive educational tools, provided that their outputs are reviewed and contextualized by healthcare professionals.

In this context, although many responses were perceived as acceptable by experts, some responses did not fully reflect the level of detail required for clinical decision-making. Consistent with the literature, large language models may generate responses that appear clinically plausible and understandable, but may still be incomplete or contextually limited in clinical settings [1,23]. One important clinical implication of this limitation is the possibility that patients may base decisions on information that appears accurate but lacks critical details. In particular, insufficient information regarding treatment options, follow-up requirements, or potential risks may lead to delays in care or inappropriate decision-making. At the same time, the ability of these systems to provide accessible and understandable information may represent an opportunity for improving patient education and awareness. From a social perspective, AI-based systems offer rapid, accessible, and comprehensible health information, which may facilitate access to medical knowledge and support health literacy. However, reliance on such systems in the presence of incomplete or contextually limited information may also contribute to the development of inappropriate health behaviors. Therefore, AI-generated information should be used as a supportive tool in patient education and should be interpreted in conjunction with professional medical guidance.

The expert opinion findings further suggest an important distinction between educational usefulness and unsupervised use. Although most experts considered the responses useful or partially useful for patient education, only a small proportion supported presenting them to patients without physician guidance. This finding should be interpreted together with the adequacy and risk findings. Most responses were perceived as harmless or low risk, but insufficient information was the most frequently reported issue, and adequacy and scientific accuracy received relatively lower ratings than clarity. Therefore, experts’ caution regarding unsupervised use may not reflect a perception of immediate harm, but rather concern that AI-generated responses may lack sufficient clinical depth, individualization, or contextual guidance for direct patient use. A response that is generally appropriate for one patient may be incomplete or misleading for another if it is applied without considering individual clinical characteristics.

In line with the DECIDE-AI framework, one of the key strengths of this study is the evaluation of responses not only in terms of accuracy but also with respect to potential clinical risk. The assessment of AI systems should consider not only accuracy but also clinical risk and safety dimensions [6,17]. The findings of this study showed that the majority of responses were classified as harmless or low risk, and no responses were identified as high risk. These results suggest that, within the evaluated question set and expert-rating framework, the responses were generally perceived as low risk. However, the presence of a limited number of responses categorized as moderate risk suggests that, in certain cases, the outputs should be interpreted with caution from a clinical perspective. This approach is consistent with studies emphasizing that AI applications should be integrated into clinical settings in a controlled and responsible manner [24,25].

Overall, this study suggests that large language models may have potential as supportive tools in patient education, provided that their outputs are interpreted with appropriate clinical contextualization and are not used as standalone sources of medical advice. These systems should therefore not be regarded as independent decision-makers, but rather as supplementary tools that may support patient education when used alongside professional medical guidance.

An important methodological consideration is the absence of a formal comparison with guideline-based reference answers. The AI-generated responses were evaluated by experts but were not directly compared with recommendations from established scoliosis guidelines or consensus documents. Therefore, high expert agreement should not be interpreted as definitive evidence of guideline concordance or complete clinical validity.

This study has several limitations. First, the evaluation was based on AI-generated responses obtained within a specific time frame. As large language model outputs may evolve over time with model updates, the findings may vary across different time points. Second, the evaluation relied on expert opinions, and the patient perspective was not directly assessed. Therefore, important dimensions such as patient understanding, emotional response, trust, and perceived usefulness could not be evaluated. Third, the study was limited to a single large language model, which may restrict the generalizability of the findings to other AI systems. Fourth, the question set was not developed using direct patient interviews, focus groups, or validated patient-information-needs instruments. Although the questions were based on publicly accessible patient-oriented scoliosis sources, they may not fully represent the diversity of real-world patient concerns. Fifth, this study focused on expert-perceived response quality and potential clinical risk, but did not assess the impact of AI-generated information on patient behavior, decision-making processes, or clinical outcomes. Sixth, the study did not include a formal gold-standard comparison. Although responses were evaluated by experienced clinicians, they were not directly compared with predefined guideline-based reference answers derived from established scoliosis recommendations. Finally, the dichotomization of Likert scores for CVR/CVI calculation and the marked ceiling effect in the rating distribution may have reduced the granularity of expert evaluations. Therefore, CVR/CVI findings were interpreted together with the original ordinal rating distributions and sensitivity analyses.

## 5. Conclusions

In this controlled expert-evaluation study, ChatGPT-5.3 responses to patient-oriented scoliosis questions were generally perceived by clinicians as understandable and appropriate within the selected question set. However, these findings reflect expert-perceived appropriateness rather than definitive evidence of factual correctness, clinical validity, or guideline concordance. Although AI-generated responses may serve as supplementary educational information, they should not be used as standalone sources of medical advice or as substitutes for individualized physician assessment. Further studies incorporating patient perspectives, guideline-based reference comparisons, and real-world outcome assessments are needed.

## Figures and Tables

**Table 1:** Patient-oriented questions on scoliosis.

Q No.	Question	Thematic Domain
Q1	What is scoliosis?	Definition/basic knowledge
Q2	What causes scoliosis?	Etiology
Q3	Does scoliosis progress over time?	Progression/complications
Q4	Can scoliosis lead to disability?	Progression/complications
Q5	What are the possible complications of scoliosis?	Progression/complications
Q6	Does scoliosis cause back or low back pain?	Symptoms
Q7	How is scoliosis diagnosed and which tests are used?	Diagnosis/screening
Q8	If there is a family history of scoliosis, when should children be screened?	Diagnosis/screening
Q9	Should siblings of a patient with scoliosis also be evaluated?	Diagnosis/screening
Q10	Can scoliosis be completely cured?	Treatment/follow-up
Q11	What is the best treatment for scoliosis?	Treatment/follow-up
Q12	Can scoliosis improve without treatment?	Treatment/follow-up
Q13	In which cases is surgery required for scoliosis?	Treatment/follow-up
Q14	Can scoliosis be corrected with exercise or physical therapy?	Treatment/follow-up
Q15	Does brace treatment stop progression?	Treatment/follow-up
Q16	How many hours per day and for how many years should a brace be worn?	Treatment/follow-up
Q17	Does carrying a heavy school bag worsen scoliosis?	Daily life/alternative beliefs
Q18	What is the most suitable sport for scoliosis?	Daily life/alternative beliefs
Q19	Can osteopathy, chiropractic, or manual therapy cure scoliosis?	Daily life/alternative beliefs
Q20	Does wearing orthodontic braces affect scoliosis?	Daily life/alternative beliefs

**Table 2:** Definitions used for clinical risk assessment.

Risk Level	Operational Definition
Harmless	General educational information without expected risk of misguidance, delayed care, or inappropriate treatment.
Low risk	Minor omission or limited detail, but unlikely to change care-seeking behavior or cause harm.
Moderate risk	Information that may create misleading reassurance, delay appropriate clinical evaluation, or lead to inappropriate self-management.
High risk	Clearly incorrect or potentially harmful recommendation that may delay urgent care, discourage necessary treatment, or promote unsafe intervention.

**Table 3:** Participant characteristics.

Variable	Value
Number of participants	51
Female, n (%)	27 (52.9%)
Male, n (%)	24 (47.1%)
Age	
Mean ± SD	37.8 ± 7.4
Min–max	27–56
Professional experience	
Mean ± SD	12.5 ± 7.7
Min–max	5–35
Profession, n (%)	
Physical Medicine and Rehabilitation (PM&R)	24 (47.1%)
Orthopedics and Traumatology	15 (29.4%)
Physiotherapist	12 (23.5%)
Experience in scoliosis assessment/treatment, n (%)	51 (100.0%)

Values are presented as mean ± standard deviation (SD) or number (percentage), unless otherwise stated.

**Table 4:** Item-level CVR values and appropriateness rates of ChatGPT-5.3 responses by question and evaluation dimension.

Question	General Appropriateness n/N (%)|CVR	Scientific Accuracy n/N (%)|CVR	Adequacy n/N (%)|CVR	Clarity n/N (%)|CVR
Q1	51/51 (100.0)|1.00	51/51 (100.0)|1.00	51/51 (100.0)|1.00	51/51 (100.0)|1.00
Q2	51/51 (100.0)|1.00	51/51 (100.0)|1.00	50/51 (98.0)|0.96	49/51 (96.1)|0.92
Q3	51/51 (100.0)|1.00	51/51 (100.0)|1.00	51/51 (100.0)|1.00	51/51 (100.0)|1.00
Q4	51/51 (100.0)|1.00	51/51 (100.0)|1.00	51/51 (100.0)|1.00	51/51 (100.0)|1.00
Q5	51/51 (100.0)|1.00	51/51 (100.0)|1.00	51/51 (100.0)|1.00	51/51 (100.0)|1.00
Q6	50/51 (98.0)|0.96	50/51 (98.0)|0.96	50/51 (98.0)|0.96	51/51 (100.0)|1.00
Q7	51/51 (100.0)|1.00	51/51 (100.0)|1.00	50/51 (98.0)|0.96	49/51 (96.1)|0.92
Q8	50/51 (98.0)|0.96	50/51 (98.0)|0.96	51/51 (100.0)|1.00	51/51 (100.0)|1.00
Q9	51/51 (100.0)|1.00	51/51 (100.0)|1.00	49/51 (96.1)|0.92	51/51 (100.0)|1.00
Q10	51/51 (100.0)|1.00	51/51 (100.0)|1.00	51/51 (100.0)|1.00	51/51 (100.0)|1.00
Q11	51/51 (100.0)|1.00	51/51 (100.0)|1.00	51/51 (100.0)|1.00	51/51 (100.0)|1.00
Q12	50/51 (98.0)|0.96	50/51 (98.0)|0.96	50/51 (98.0)|0.96	51/51 (100.0)|1.00
Q13	51/51 (100.0)|1.00	51/51 (100.0)|1.00	51/51 (100.0)|1.00	51/51 (100.0)|1.00
Q14	51/51 (100.0)|1.00	51/51 (100.0)|1.00	51/51 (100.0)|1.00	51/51 (100.0)|1.00
Q15	51/51 (100.0)|1.00	51/51 (100.0)|1.00	51/51 (100.0)|1.00	51/51 (100.0)|1.00
Q16	50/51 (98.0)|0.96	50/51 (98.0)|0.96	50/51 (98.0)|0.96	51/51 (100.0)|1.00
Q17	51/51 (100.0)|1.00	51/51 (100.0)|1.00	51/51 (100.0)|1.00	51/51 (100.0)|1.00
Q18	49/51 (96.1)|0.92	48/51 (94.1)|0.88	48/51 (94.1)|0.88	50/51 (98.0)|0.96
Q19	51/51 (100.0)|1.00	51/51 (100.0)|1.00	51/51 (100.0)|1.00	51/51 (100.0)|1.00
Q20	50/51 (98.0)|0.96	50/51 (98.0)|0.96	51/51 (100.0)|1.00	51/51 (100.0)|1.00
CVI	0.99	0.98	0.98	0.99

Appropriate percentages and CVR values were calculated after dichotomizing ratings as 1–3 = inappropriate and 4–6 = appropriate. CVI was calculated as the average CVR across the 20 questions for each dimension. CVR: Content Validity Ratio; CVI: Content Validity Index. CVR and CVI values ≥0.26 were considered acceptable based on Lawshe’s criteria.

**Table 5:** Sensitivity analysis using original 6-point ordinal ratings.

Question	General Appropriateness Mean ± SD (Median [IQR])	Scientific Accuracy Mean ± SD (Median [IQR])	Adequacy Mean ± SD (Median [IQR])	Clarity Mean ± SD (Median [IQR])
Q1	4.90 ± 1.01 (4.0 [2.0])	4.94 ± 1.01 (4.0 [2.0])	4.94 ± 1.01 (4.0 [2.0])	5.33 ± 0.95 (6.0 [2.0])
Q2	4.24 ± 0.65 (4.0 [0.0])	4.35 ± 0.77 (4.0 [0.0])	4.25 ± 0.72 (4.0 [0.0])	4.63 ± 1.00 (4.0 [2.0])
Q3	5.45 ± 0.90 (6.0 [2.0])	5.41 ± 0.92 (6.0 [2.0])	5.41 ± 0.92 (6.0 [2.0])	5.61 ± 0.80 (6.0 [0.0])
Q4	5.65 ± 0.77 (6.0 [0.0])	5.61 ± 0.80 (6.0 [0.0])	5.57 ± 0.83 (6.0 [0.0])	5.61 ± 0.80 (6.0 [0.0])
Q5	5.49 ± 0.88 (6.0 [1.0])	5.53 ± 0.86 (6.0 [0.0])	5.41 ± 0.92 (6.0 [2.0])	5.53 ± 0.86 (6.0 [0.0])
Q6	5.47 ± 0.92 (6.0 [1.0])	5.39 ± 0.96 (6.0 [2.0])	5.39 ± 0.96 (6.0 [2.0])	5.61 ± 0.80 (6.0 [0.0])
Q7	4.94 ± 1.01 (4.0 [2.0])	4.98 ± 1.01 (4.0 [2.0])	4.80 ± 1.02 (4.0 [2.0])	4.47 ± 0.92 (4.0 [1.0])
Q8	4.84 ± 1.03 (4.0 [2.0])	4.84 ± 1.03 (4.0 [2.0])	4.78 ± 0.99 (4.0 [2.0])	4.90 ± 1.01 (4.0 [2.0])
Q9	4.51 ± 0.88 (4.0 [1.0])	4.43 ± 0.83 (4.0 [0.0])	4.35 ± 0.84 (4.0 [0.0])	4.71 ± 0.97 (4.0 [2.0])
Q10	5.53 ± 0.86 (6.0 [0.0])	5.53 ± 0.86 (6.0 [0.0])	5.49 ± 0.88 (6.0 [1.0])	5.53 ± 0.86 (6.0 [0.0])
Q11	5.18 ± 0.99 (6.0 [2.0])	5.14 ± 1.00 (6.0 [2.0])	5.14 ± 1.00 (6.0 [2.0])	5.37 ± 0.94 (6.0 [2.0])
Q12	5.24 ± 1.01 (6.0 [2.0])	5.20 ± 1.02 (6.0 [2.0])	5.24 ± 1.01 (6.0 [2.0])	5.41 ± 0.92 (6.0 [2.0])
Q13	5.10 ± 1.01 (6.0 [2.0])	5.06 ± 1.01 (6.0 [2.0])	5.14 ± 1.00 (6.0 [2.0])	5.06 ± 1.01 (6.0 [2.0])
Q14	5.49 ± 0.88 (6.0 [1.0])	5.49 ± 0.88 (6.0 [1.0])	5.53 ± 0.86 (6.0 [0.0])	5.57 ± 0.83 (6.0 [0.0])
Q15	5.22 ± 0.99 (6.0 [2.0])	5.18 ± 0.99 (6.0 [2.0])	5.22 ± 0.99 (6.0 [2.0])	5.37 ± 0.94 (6.0 [2.0])
Q16	5.00 ± 1.04 (6.0 [2.0])	4.96 ± 1.04 (4.0 [2.0])	5.12 ± 1.03 (6.0 [2.0])	5.10 ± 1.01 (6.0 [2.0])
Q17	4.75 ± 0.98 (4.0 [2.0])	4.75 ± 0.98 (4.0 [2.0])	4.75 ± 0.98 (4.0 [2.0])	5.10 ± 1.01 (6.0 [2.0])
Q18	4.47 ± 0.92 (4.0 [1.0])	4.41 ± 0.92 (4.0 [0.0])	4.45 ± 0.94 (4.0 [1.0])	4.88 ± 1.03 (4.0 [2.0])
Q19	5.06 ± 1.01 (6.0 [2.0])	5.06 ± 1.01 (6.0 [2.0])	5.06 ± 1.01 (6.0 [2.0])	4.98 ± 1.01 (4.0 [2.0])
Q20	4.45 ± 0.88 (4.0 [0.0])	4.49 ± 0.90 (4.0 [1.0])	4.51 ± 0.88 (4.0 [1.0])	4.59 ± 0.92 (4.0 [2.0])

Data are presented as mean ± standard deviation (median [interquartile range]). Values were calculated using the original 1–6 ordinal Likert ratings without dichotomization. Each question–dimension cell included 51 expert ratings. Higher scores indicate more favorable expert evaluations. SD: standard deviation; IQR: interquartile range.

**Table 6:** Reasons for inappropriate general appropriateness.

Reasons	n (%)
Contains insufficient information	12 (%52.2)
Contains scientific inaccuracies	5 (%21.7)
Has language or clarity issues	2 (%8.7)
Contains excessive or unnecessary information	2 (%8.7)
Does not adequately answer the question/is off-topic	1 (%4.3)
Other	1 (%4.3)

Values are presented as number (percentage).

**Table 7:** Most commonly reported overall issue by experts (n = 51).

Most Common Issue	N (%)
Contains insufficient information	24 (%47.1)
No error	18 (%35.3)
Does not fully answer the question/off-topic	4 (%7.8)
Contains excessive/unnecessary information	3 (%5.9)
Contains scientific error	2 (%3.9)
Language or expression issues	0
Other	0

Values are presented as number (percentage) of responses.

## Data Availability

The datasets generated and/or analyzed during the current study, including the question set, AI-generated responses, and expert evaluation data, are available from the author upon reasonable request.

## References

[1] Sallam M. (2023). ChatGPT Utility in Healthcare Education, Research, and Practice: Systematic Review on the Promising Perspectives and Valid Concerns. Healthcare.

[2] Younis H.A., Eisa T.A.E., Nasser M., Sahib T.M., Noor A.A., Alyasiri O.M., Salisu S., Hayder I.M., Younis H.A.K. (2024). A Systematic Review and Meta-Analysis of Artificial Intelligence Tools in Medicine and Healthcare: Applications, Considerations, Limitations, Motivation and Challenges. Diagnostics.

[3] Garg R.K., Urs V.L., Agarwal A.A., Chaudhary S.K., Paliwal V., Kar S.K. (2023). Exploring the Role of ChatGPT in Patient Care (Diagnosis and Treatment) and Medical Research: A Systematic Review. Health Promot. Perspect..

[4] Xu X., Chen Y., Miao J. (2024). Opportunities, Challenges, and Future Directions of Large Language Models, Including ChatGPT in Medical Education: A Systematic Scoping Review. J. Educ. Eval. Health Prof..

[5] Eysenbach G. (2023). The Role of ChatGPT, Generative Language Models, and Artificial Intelligence in Medical Education: A Conversation With ChatGPT and a Call for Papers. JMIR Med. Educ..

[6] Wang L., Wan Z., Ni C., Song Q., Li Y., Clayton E., Malin B., Yin Z. (2024). Applications and Concerns of ChatGPT and Other Conversational Large Language Models in Health Care: Systematic Review. J. Med. Internet Res..

[7] Preiksaitis C., Rose C. (2023). Opportunities, Challenges, and Future Directions of Generative Artificial Intelligence in Medical Education: Scoping Review. JMIR Med. Educ..

[8] Zhang C., Liu S., Zhou X., Zhou S., Tian Y., Wang S., Xu N., Li W. (2024). Examining the Role of Large Language Models in Orthopedics: Systematic Review. J. Med. Internet Res..

[9] Sharma A., Amerio T., Etemadimanesh A., Ghasemi A., Hai B., Lodhi S., Malja E., Pennington C.C., Xi Y., He A. (2025). The Utility of ChatGPT in Musculoskeletal Imaging-Related Patient Education. Clin. Imaging.

[10] Cordani C., Malisano L., Febbo F., Giranio G., Del Furia M.J., Donzelli S., Negrini S. (2023). Influence of Specific Interventions on Bracing Compliance in Adolescents with Idiopathic Scoliosis—A Systematic Review of Papers Including Sensors’ Monitoring. Sensors.

[11] Fazalbhoy A., McAviney J., Mirenzi R. (2025). Compliance of Physiotherapeutic Scoliosis-Specific Exercise in Adolescent Idiopathic Scoliosis: A Scoping Review. J. Clin. Med..

[12] AlSamhori J.F., Alkafaween A.M., Al-Badawi A.W.A., Alhabashneh Z.T., Alelaumi A.F., Haddad B.I., Nashwan A.J. (2025). The Role of ChatGPT in Improving Orthopedic Patient Education in Low-Resource Settings across Various Orthopedic Specialties. J. Precis. Med. Health Dis..

[13] Johnson D., Goodman R., Patrinely J., Stone C., Zimmerman E., Donald R., Chang S., Berkowitz S., Finn A., Jahangir E. (2023). Assessing the Accuracy and Reliability of AI-Generated Medical Responses: An Evaluation of the Chat-GPT Model. Res. Sq. Res. Sq. [Prepr.].

[14] Giray E., Korkmaz M.D., Illeez O.G., Capan N., Karadag Saygi E., Aydin A.R. (2025). Let’s Chat (GPT) about Adolescent Idiopathic Scoliosis: Accuracy and Reliability of Chat Responses to Frequently Asked Questions. BMC Musculoskelet. Disord..

[15] Negrini F., Malfitano C., Ferriero G., Morone G., Negrini A., Zaina F., Ferrario I., Kiekens C., Negrini S., Vitale J. (2025). Evaluating ChatGPT-4.0’s Accuracy and Potential in Idiopathic Scoliosis Conservative Treatment: A Preliminary Study on Clarity, Validity, and Expert Perceptions. Eur. Spine J..

[16] Republic of Türkiye (2016). Personal Data Protection Law (Law No. 6698).

[17] Vasey B., Nagendran M., Campbell B., Clifton D.A., Collins G.S., Denaxas S., Denniston A.K., Faes L., Geerts B., Ibrahim M. (2023). Reporting Guideline for the Early Stage Clinical Evaluation of Decision Support Systems Driven by Artificial Intelligence: DECIDE-AI. BMJ.

[18] Lawshl C.H. (1975). A Quantitative Approach to Content Validity. Pers. Psychol..

[19] Gilson A., Safranek C.W., Huang T., Socrates V., Chi L., Taylor R.A., Chartash D. (2023). How Does ChatGPT Perform on the United States Medical Licensing Examination? The Implications of Large Language Models for Medical Education and Knowledge Assessment. JMIR Med. Educ..

[20] Kung T.H., Cheatham M., Medenilla A., Sillos C., De Leon L., Elepaño C., Madriaga M., Aggabao R., Diaz-Candido G., Maningo J. (2023). Performance of ChatGPT on USMLE: Potential for AI-Assisted Medical Education Using Large Language Models. PLoS Digit. Health.

[21] Huh S. (2023). Are ChatGPT’s Knowledge and Interpretation Ability Comparable to Those of Medical Students in Korea for Taking a Parasitology Examination?: A Descriptive Study. J. Educ. Eval. Health Prof..

[22] Zec S., Soriani N., Comoretto R., Baldi I. (2017). High Agreement and High Prevalence: The Paradox of Cohen’s Kappa. Open Nurs. J..

[23] Bender E.M., Gebru T., McMillan-Major A., Shmitchell S. (2021). On the Dangers of Stochastic Parrots: Can Language Models Be Too Big?. Proceedings of the FAccT 2021—2021 ACM Conference on Fairness, Accountability, and Transparency.

[24] Topol E.J. (2019). High-Performance Medicine: The Convergence of Human and Artificial Intelligence. Nat. Med..

[25] Esteva A., Robicquet A., Ramsundar B., Kuleshov V., DePristo M., Chou K., Cui C., Corrado G., Thrun S., Dean J. (2019). A Guide to Deep Learning in Healthcare. Nat. Med..

